# Effects of first‐line antidiabetic drugs on the improvement of arterial stiffness: A Bayesian network meta‐analysis

**DOI:** 10.1111/1753-0407.13405

**Published:** 2023-05-10

**Authors:** Jincheng Wang, Yuhan Wang, Yueheng Wang, Yu Li, Jiamei Zhang, Han Zhang, Xiaomin Fu, Zhiqin Guo, Ying Yang, Kaining Kang, Wei Zhang, Li Tian, Yanqiang Wu, Shuanli Xin, Hongzhou Liu

**Affiliations:** ^1^ Department of Epidemiology The George Washington University Washington DC USA; ^2^ Department of Endocrinology Beijing Friendship Hospital, Capital Medical University Beijing China; ^3^ Department of Ultrasound Diagnosis The Second Hospital of Hebei Medical University Shijiazhuang China; ^4^ Department of General Internal Medicine The First Affiliated Hospital of Sun Yat‐sen University Guangzhou China; ^5^ Cardiovascular department The First Hospital of Tsinghua University Beijing China; ^6^ Department of Geriatric Diseases Handan Central Hospital Handan China; ^7^ Department of Cardiology First Hospital of Handan City Handan China; ^8^ Department of Endocrinology First Hospital of Handan City Handan China

**Keywords:** antidiabetic drugs, arterial stiffness, cardiovascular outcome, pulse wave velocity, vascular function, 降糖药物, 动脉硬化, 脉搏波传导速度, 血管功能, 心血管结局

## Abstract

**Background:**

Changes in vascular function are closely associated with the development of cardiovascular disease (CVD). Pulse wave velocity (PWV) is a potential indicator of vascular dysfunction; it allows noninvasive assessment of arterial stiffness. Currently, evidence for the effects of different classes of antidiabetic drugs on arterial stiffness remains limited. In this study, a network meta‐analysis (NMA) was performed to explore the associations between changes in arterial stiffness and first‐line antidiabetic drugs by evaluating PWV in patients with different metabolic abnormalities.

**Methods:**

We systematically searched several electronic databases for randomized controlled trials (RCTs) published from inception until 25 August 2022, without language restrictions. The primary outcome was the change in PWV (ΔPWV) in all included studies; subgroup analysis was performed for patients with abnormal glucose metabolism, including prediabetes and diabetes mellitus. NMA was performed to calculate the mean differences (MDs) with 95% confidence intervals (CIs) as effect sizes to evaluate the ΔPWV.

**Results:**

Among the 2257 candidate articles identified in the initial search, 18 RCTs were eventually included in the analysis. In all studies, two classes of new antidiabetic drugs, glucagon‐like peptide‐1 receptor (GLP‐1R) agonists and sSodium‐glucose co‐transporter 2 (SGLT‐2) inhibitors, improved arterial stiffness by decreasing PWV compared with placebo (MD = −1.11, 95% CI: −1.94 to 0.28) and (MD = −0.76, 95% CI: −1.45 to −0.08). A conventional antidiabetic drug, metformin, also showed similar efficacy compared with placebo (MD = −0.73, 95% CI: −1.33 to −0.12). Finally, in subgroup studies of patients with abnormal glucose metabolism diseases, GLP‐1R agonists (MD = −1.06, 95% CI: −2.05 to −0.10) significantly decreased PWV compared with placebo.

**Conclusion:**

Three classes of antidiabetic drugs—GLP‐1R agonists, SGLT‐2 inhibitors, and metformin—have the potential to improve arterial stiffness. Among the six classes of antidiabetic drugs analyzed, GLP‐1R agonists constitute the only class of drugs that improves arterial stiffness in patients with abnormal glucose metabolism diseases.

## Graphical abstract

**Highlights**

- Vascular complication is one of the most important causes of death in diabetes patients.
- Arterial stiffness plays a key role in the progress of diabetes vascular complication; therefore, the improvement of arterial stiffness could inhibit the process of atherosclerosis and reduce the burden of diabetes vascular complications
- This study first analyzed the potential antiarterial stiffness effect of six first‐line antidiabetic drugs, providing new strategies for personalized glucose‐lowering therapy.

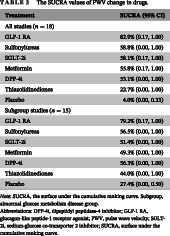

## 摘要

背景:血管功能的改变与心血管疾病(CVD)的发展密切相关。脉搏波传导速度(PWV)是血管功能紊乱的潜在指标, 可无创评估动脉硬化程度。目前, 不同类型的降糖药对动脉硬化影响的证据仍然有限。本研究通过网络meta分析(NMA)评估不同代谢异常患者的PWV, 探讨动脉硬化变化与一线降糖药物的关系。

方法:系统检索多个电子数据库, 纳入从建库至2022年8月25日发表的随机对照试验(RCTs), 无语言限制。主要结局指标为所有纳入研究中PWV的变化(ΔPWV);对糖代谢异常患者进行亚组分析, 包括糖尿病前期和糖尿病。采用NMA计算均数差(MDs)及其95%可信区间(CIs)作为效应量评价ΔPWV。

结果:共检索到2257篇文献, 最终纳入18项RCTs进行分析。在所有研究中, 与安慰剂相比, 两类新型降糖药物, GLP‐1受体激动剂和SGLT‐2抑制剂通过降低PWV改善动脉硬化(MD =−1.11,95% CI:−1.94 ~ 0.28)和(MD =−0.76,95% CI:−1.45 ~−0.08), 以及传统降糖药二甲双胍也显示出相似的疗效(MD = ‐ 0.73, 95% CI: ‐ 1.33 ~ ‐ 0.12)。最后, 在糖代谢异常疾病患者的亚组研究中, 与安慰剂相比, GLP‐1受体激动剂(MD =−1.06,95% CI:−2.05 ~−0.10)显著降低了PWV。

结论:三类降糖药物, 即GLP‐1受体激动剂, SGLT‐2抑制剂和二甲双胍, 具有改善动脉硬化的潜力。在分析的六类降糖药物中, GLP‐1受体激动剂是唯一一类可以改善异常糖代谢患者动脉硬化的药物。

## 1 INTRODUCTION

Cardiovascular complication is one of the main reasons for patients with diabetes to experience disability and death. Recently, more studies are paying attention on the cardiovascular effect of antidiabetic drugs. In two large‐scale trials, LEADER^1^ and EMPA‐REG OUTCOME,^2^ vascular‐related outcomes such as cardiovascular diseases (CVDs), cardiovascular mortality, and other cardiovascular complications of diabetes were regarded as the main outcomes for evaluating the comprehensive effects of hypoglycemic drugs, with a particular focus on newer antidiabetic drugs. Several novel antidiabetic drugs have shown benefits for CVD in type 2 diabetes mellitus (T2DM) patients. For example, the glucagon‐like peptide‐1 receptor (GLP‐1R) agonists may reduce the risk of major cardiovascular events, whereas sodium‐glucose co‐transporter 2 (SGLT‐2) inhibitors decreased the risks of dialysis and kidney disease‐related death, in T2DM patients.^3^, ^4^ In addition, our previous research has proved the positive effect of GLP‐1R agonists on endothelial function.^4^, ^5^ However, the effects of different antidiabetic drugs on other aspects of vascular function have not yet been extensively investigated.

Arterial stiffness can sensitively reflect the changes of arterial function in individuals. Arterial stiffness is a consequence of vascular aging and has a strong association with the risk of CVD. Pulse wave velocity (PWV) has been considered the gold standard for assessing arterial stiffness^5^; PWV values serve as noninvasive and simple measurements of arterial elasticity. Higher PWV values represent arterial wall dysfunction and structural damage. PWV is a potential indicator that can be used as a clinical predictor of long‐term cardiovascular risk.^6^

The effects of new antidiabetic drugs on change in PWV (ΔPWV) have been explored in several studies^7^, ^8^; however, there is limited evidence regarding different classes of hypoglycemic drugs. The utility of conventional pairwise meta‐analysis is limited because the effects of treatments in head‐to‐head trials cannot be evaluated. The network meta‐analysis (NMA) method has overcome this limitation because it allows comparison of the effects of ≥2 treatments through direct and indirect evidence.^9^ Here, we performed an NMA and systematic review of randomized controlled trials (RCTs) to comprehensively explore the effects of different antidiabetic drugs on arterial stiffness (represented by PWV).

## 2 METHODS

### 2.1 Data sources and searches

This study was conducted in accordance with the Network Meta‐analysis of the Preferred Reporting Items for Systematic Reviews and Meta‐Analyses (PRISMA‐NMA) statement and the Cochrane Handbook for Systematic Reviews. Two reviewers (JCW and YHW) initially screened titles and abstracts independently; they subsequently reviewed the full texts of potentially eligible articles to determine suitability for inclusion in the analysis. Disagreements between the two reviewers were resolved by discussion; if necessary, a third reviewer with additional clinical experience (HZL) was consulted. Because all analyses were based on previous published studies, there were no requirements for ethical approval or patient consent. PubMed, Embase, and the Cochrane Central Register of Controlled Trials were searched from inception to 25 August 2022, to identify potentially eligible RCTs in any language. The following Medical Subject Headings (MeSH) terms and free text terms, combined with Boolean operators, were used in the search strategy: “hypoglycemic agents,” “sodium‐glucose transporter 2 inhibitors,” “glucagon‐like peptide‐1,” “dipeptidyl‐peptidase IV inhibitors,” “thiazolidinedione,” “α‐glucosidase inhibitors,” “glinides,” “metformin,” “sulfonylureas,” “arterial stiffness,” “pulse wave analysis,” and “randomized controlled trials.” Additionally, a recursive manual search was conducted to retrieve full texts of studies from the bibliographies of relevant reports or similar systematic reviews; this strategy was used to identify potentially eligible studies that may have been missed in the initial review process. The details of the search strategy are presented in the Supplementary Material. All citations were managed using Endnote X9 software (Thompson ISI Research Soft, Philadelphia, PA, USA).

### 2.2 Study selection

RCTs were included if they met the PICOS criteria; these RCTs are summarized here.

### 2.3 Population

To increase comparability among studies, the analysis included participants in studies with ≥1 of the following metabolism‐related diseases: coronary artery disease, chronic heart failure, type 1 diabetes mellitus (T1DM), T2DM, and nonalcoholic fatty liver disease (NAFLD). The pre‐T2DM, T1DM, T2DM were summarized as abnormal glucose metabolism diseases (AGMD).

### 2.4 Treatment

Six classes of antidiabetic drugs were included in the analysis: SGLT‐2 inhibitors, GLP‐1R agonists, dipeptidyl peptidase 4 (DPP‐4) inhibitors, thiazolidinediones, metformin, and sulfonylureas. Treatments were excluded if they involved agents withdrawn by the Food and Drug Administration, such as rosiglitazone.

### 2.5 Comparison

Treatments involving the six classes of antidiabetic drugs were compared with each other or with placebo.

### 2.6 Study design

The analysis was confined to published RCTs without restrictions on year or language. Other studies, such as single‐arm trials, were excluded.

### 2.7 Outcome

The outcome was arterial stiffness, which was defined as ΔPWV from baseline to post treatment. The primary outcome was PWV change in all studies. The subgroup outcome was PWV change in patients with AGMD.

### 2.8 Data extraction and quality assessment

Two authors (JCW and YHW) independently extracted relevant data from the included studies by using the Cochrane Consumers and Communication Review Group's data extraction template. The extracted data included first author name, publication year, participant baseline characteristics (treatment, sample size, baseline age, baseline body mass index, and baseline HbA1c level), and RCT quality. The ΔPWV from baseline to post treatment was either directly extracted or calculated from the mean ± SD PWV values, combined with the numbers of patients at baseline and at the last observation. Mean differences and 95% confidence intervals (CIs) are presented for the effect sizes of ΔPWV. Risk of bias was evaluated using the Cochrane Risk of Bias tool. Any discrepancies in data extraction or quality assessment were resolved through discussion with the third author (HZL).

### 2.9 Data synthesis and analysis

A conventional pairwise meta‐analysis was initially conducted to analyze direct evidence from the included studies. The heterogeneity of treatment effects across trials was assessed using *I* ^2^ statistics. In cases where the *p* value was ≥.1 and the *I* ^2^‐value was ≤50%, statistical heterogeneity was considered mild. In cases where the *p* value was <.1 and the *I* ^2^‐value was >50%, sources of heterogeneity were investigated using subgroup analysis or meta‐regression. Comparison‐adjusted funnel plots were drawn to determine the presence of publication bias.

Because the presence of an effect size implies a continuous outcome, pooled mean difference values and 95% CIs were generated to summarize the effect sizes. For each comparison, pooled mean differences were calculated using the changes in group mean and SD in each study. If a change in SD was not available for particular study, the following formula was used to calculate the change in SD from baseline for experimental and comparator treatments: SD change = [baselineSD^2^ + finalSD^2^ − (2 × Corr × baselineSD × final SD)]^1/2^.^10^ If the extracted mean and SD data were presented in terms of ranges or interquartile ranges, the median was used as a substitute for the mean,^10^ range/6 was used to estimate SD,^20^ and the formula SD = (Q3−Q1)/1.35 was used to estimate SD from interquartile range.^11^

To ensure sufficient similarity in the various treatment comparisons and provide valid indirect inferences, two authors (JCW and YHW) independently evaluated the transitivity assumption before statistical analyses by comparing and examining the clinical and methodological characteristics (eg, participant characteristics, experimental design, and end point measurements) for each included study.^12^

Similar with the method in our recently published study,^45^ the Markov chain Monte Carlo method with prior noninformative distributions was used in our NMA based on the maximum likelihood and Bayesian estimation results. A random‐effects model with vague priors was used for multi‐arm trials. Normal prior distributions with a mean of 0 and a variance of 10^−4^ were used for all trial baselines and treatment effects; uniform prior distributions with a mean of 0 and a variance of 5 were used to calculate between‐trial SDs. The posterior distribution was estimated by the Markov chain Monte Carlo method using Gibbs sampling. Three parallel Markov chains were used to assess convergence by starting the analysis from different initial states (via stimulation) to obtain the target distributions.^13^ A burn‐in of 20 000 iterations was conducted to ensure that the three chains had converged; the subsequent 80 000 iterations were sampled for analysis.

The surface under the cumulative ranking curve (SUCRA) was presented as a simple numerical statistical cumulative ranking probability plot, which was summarized to evaluate the ΔPWV in each treatment. A higher SUCRA value (up to 0) indicated a greater likelihood that the treatment was highly effective, whereas a lower value (down to 0) indicated that the treatment effect was poor.^14^ Global and loop inconsistency were used to explore the consistency of direct and indirect evidence in the network, and *p* < 0.05 was considered indicative of inconsistency. All analyses were conducted in STATA, version 14.0 (StataCorp, College Station, TX, USA) and OpenBUGS, Version 3.2.3 (MRC Biostatistics Unit, Cambridge, UK).

## 3 RESULTS

### 3.1 Study selection

**FIGURE 1:**
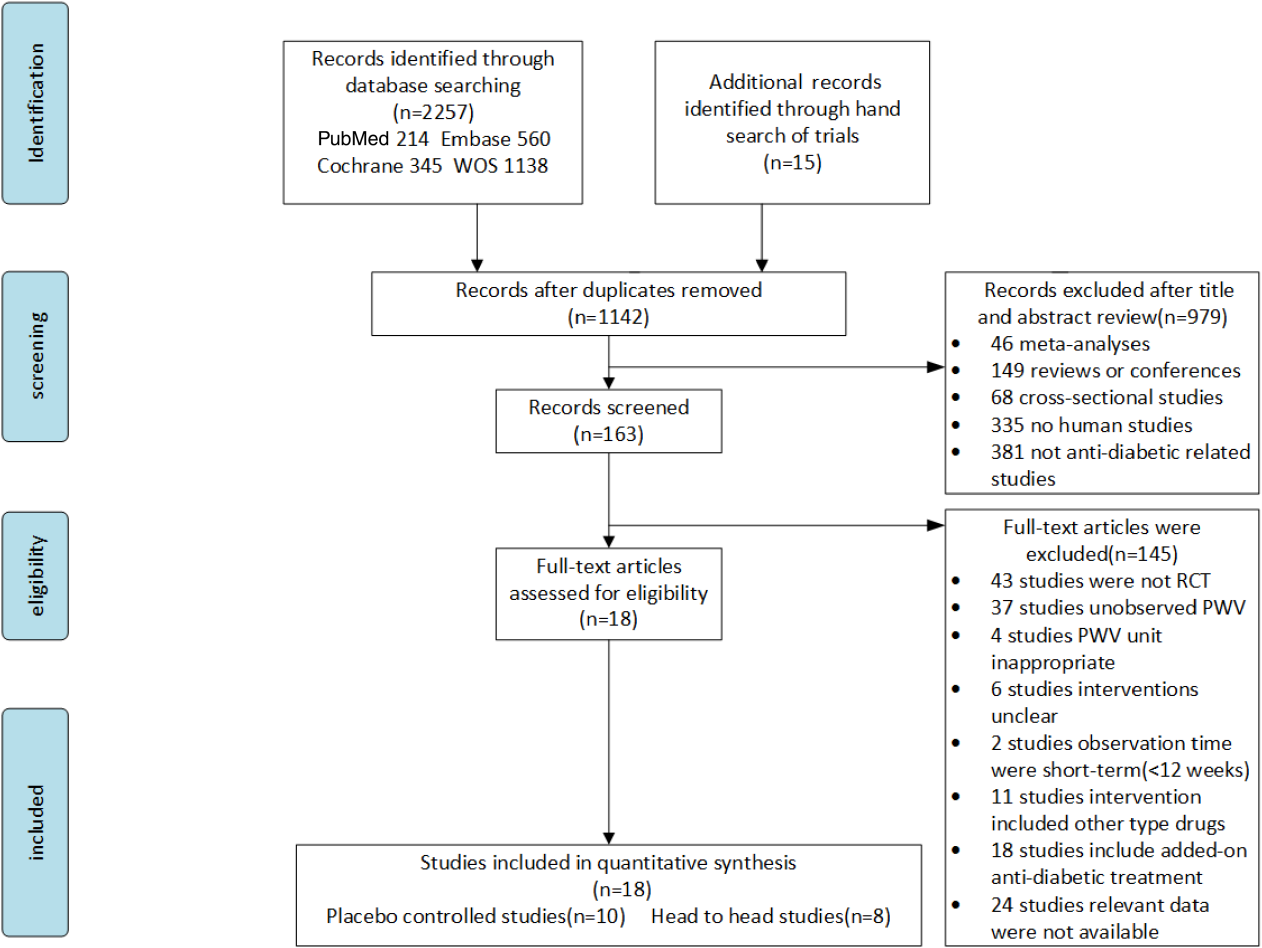
Study selection flow chart. PWV, pulse wave velocity; RCT, randomized controlled trials; WOS, World of Science.

Figure 1 shows the literature selection procedure. The initial search revealed 2257 candidate articles, including 15 articles identified by hand searches of relevant reviews or meta‐analyses; of these articles, 1130 were discarded because of duplication, 979 were removed after assessment of the title and abstract, and 145 were excluded at the full‐text stage based on exclusion criteria. After assessment of the full text, 18 articles were included in the analysis. The reasons for article exclusion are shown in Figure 1.

**FIGURE 2:**
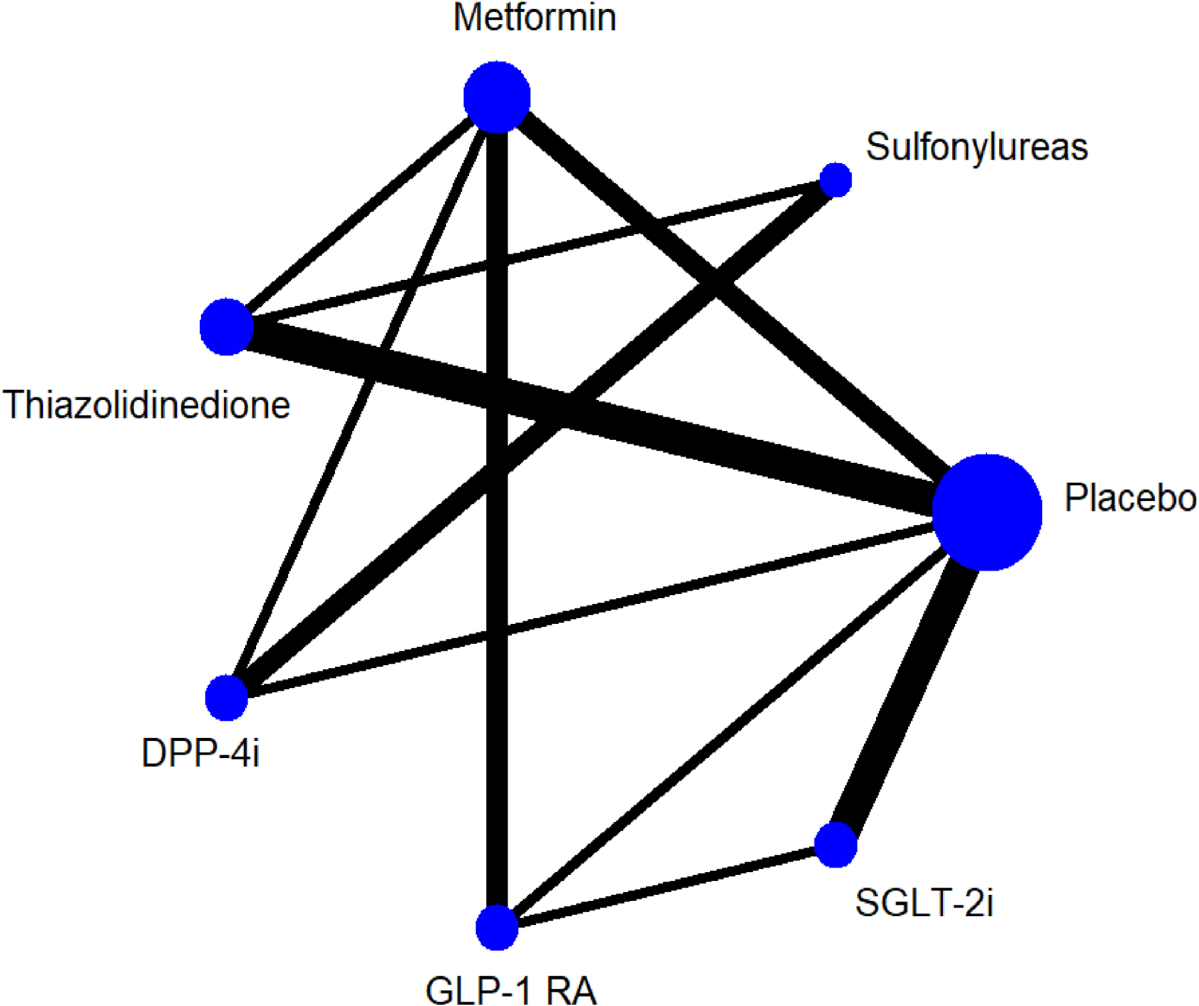
Network plot for all studies. DPP‐4i, dipeptidyl peptidase‐4 inhibitor; GLP‐1 RA, glucagon‐like peptide‐1 receptor agonist; SGLT‐2i, sodium‐glucose co‐transporter 2 inhibitor.

The 18 studies included 10 placebo‐controlled studies and 8 head‐to‐head studies. The abnormal glucose metabolism group consisted of 15 studies in which participants were diagnosed with pre‐T2DM, T1DM, or T2DM.^16^, ^17^, ^18^, ^19^, ^20^, ^21^, ^22^, ^23^, ^24^, ^25^, ^26^, ^28^, ^29^, ^30^, ^32^ Three studies included nonabnormal glucose metabolism participants, who were diagnosed with coronary artery disease, chronic heart failure, or NAFLD respectively.^15^, ^27^, ^31^ The participant characteristics and treatments are summarized in Table 1. A network plot of each treatment was produced as a summary description to provide all available evidence for each treatment (Figure 2).

**TABLE 1:** Baseline characteristics of all studies.

First author, year	Treatment		Sample size (EG/CG)	Age (years) (EG/CG) (mean ± SD)	Male (%)	Baseline HbA1c (%) (mean ± SD)	BMI (kg/m^2^) (mean ± SD)	Treatment duration (week)	Country	Baseline PWV (EG/CG, m/s) (mean ± SD)	ΔPWV (EG/CG) (mean ± SD)	PWV measure time frame (pre‐, post treatment)	Subjects characteristic
	Experience group	Control group											
Christoph^15^	TZD (Pioglitazone)	Placebo	27/27	59.5 ± 10.4/62.2 ± 10.0	0.8	5.7 ± 0.4/5.7 ± 0.5	27.7 ± 3.7/27.7 ± 3.2	36	Germany	12.9 ± 3.5/13.9 ± 3.6	0.7 ± 2.1/0.5 ± 2.1	0, 36 weeks	CAD
Antonio^16^	SGLT‐2i (Empagliflozin)	Placebo	42/42	64.2 ± 10.9/59.9 ± 13.1	0.6	NR	NR	24	USA	6.2 ± 1.6/6.0 ± 1.0	−0.6 ± 1.4/0.6 ± 1.2	0, 24 weeks	T2DM
Ikonomidis^17^	GLP‐1 RA (Liraglutide)	SGLT‐2i (Empagliflozin)	40/40	57 ± 9/58 ± 10	0.7	8 ± 1.1/7.8 ± 0.9	30 ± 4/29.8 ± 3	48	Greece	11.6 ± 2.8/12 ± 2.8	−1.1 ± 2.4/−1.1 ± 2.5	0, 48 weeks	T2DM
Stakos^18^	TZD (Troglitazone)	Placebo	40/97	40.0 ± 7.5/41.0 ± 7.0	0.2	NR	NR	48	USA	NR	1.1 ± 0.4/0.7 ± 0.4	0, 48 weeks	T2DM
Lambadiari^19^	GLP‐1 RA (Liraglutide)	Metformin	30/30	51 ± 10/50 ± 12	0.7	8.6 ± 2/7 ± 1.2	32.9 ± 5/27.7 ± 2	24	Greece	11.8 ± 2.5/10.3 ± 3.3	−0.6 ± 2.8/−0.2 ± 3.2	0, 24 weeks	T2DM
Tuttolomondo^20^	GLP‐1 RA (Dulaglutide)	Metformin	56/56	69.7 ± 8.6/67.6 ± 5.1	0.4	7.4 ± 0.7/7.2 ± 0.6	27.6 ± 3.4/27.9 ± 3.2	36	Italy	11.2 ± 0.9/10.9 ± 0.8	−0.3 ± 0.8/0.1 ± 0.7	0, 36 weeks	T2DM
Kato^21^	TZD (Pioglitazone)	Metformin	25/25	51.4 ± 15.2/58.6 ± 12.4	0.5	7.4 ± 1.8/7.1 ± 1.4	28.4 ± 6.4/27.5 ± 3.4	12	Japan	15.4 ± 0.4/14.7 ± 0.4	0.08 ± 0.4/0.01 ± 0.4	0, 12 weeks	T2DM
Papadopoulou^22^	SGLT‐2i (Dapagliflozin)	Placebo	43/42	61.7 ± 6.7/60.6 ± 9.4	0.5	7.8 ± 0.6/7.8 ± 0.4	31.33 ± 4.50/31.83 ± 7.08	12	Greece	8.8 ± 1.1/8.7 ± 1.3	−0.2 ± 1.1/0.01 ± 1.3	0, 12 weeks	T2DM
deBoer^23^	DPP‐4i (Linagliptin)	Placebo	22/22	63 ± 10.4/62 ± 9.6	0.6	6.3 ± 0.4/6.2 ± 0.5	32.3 ± 4.7/30.4 ± 6.2	26	Netherlands	8.7 ± 0.3/8.8 ± 0.3	−0.4 ± 0.3/0.4 ± 0.3	0, 26 weeks	T2DM
Kolwelter^24^	DPP‐4i (Empagliflozin)	Placebo	48/26	66 ± 9	0.9	5.8 ± 0.6/5.9 ± 0.8	28.7 ± 3.9/28.9 ± 3.3	12	Germany	9.6 ± 1.5/9 ± 1.6	−0.3 ± 1.6/0.3 ± 1.7	0, 12 weeks	CHF
Zografou^25^	DPP‐4i (Vildagliptin)	Metformin	32/32	52 ± 11.2/56 ± 10.5	0.6	8.1 ± 0.8/8 ± 0.8	31.6 ± 4.6/32.2 ± 5.9	24	Greece	8.6 ± 2.1/8.9 ± 2	−0.3 ± 1.5/0.2 ± 1.8	0, 24 weeks	T2DM
Paiman^26^	GLP‐1 RA (Liraglutide)	Placebo	22/25	55 ± 11/55 ± 9	0.4	8.1 ± 0.9/8.6 ± 1.1	30.4 ± 3.8/28.6 ± 4.0	26	Netherlands	8.8 ± 2.4/8.3 ± 2.4	0.2 ± 2.1/−0.2 ± 1.7	0, 26 weeks	T2DM
Kim^27^	TZD (Rosiglitazone)	Placebo	45/40	54.2 ± 11.9/53.4 ± 9.8	0.6	5.8 ± 0.4/5.8 ± 0.4	27.1 ± 3.2/26.2 ± 2.8	12	Korea	14.8 ± 2.6/14.4 ± 2.3	−0.9 ± 2.5/0.02 ± 2.0	0, 12 weeks	Pre‐T2DM
Bjornstad^28^	Metformin	Placebo	24/21	17.3 ± 2.3/15.9 ± 2.7	0.5	NR	NR	12	USA	NR	−1.1 ± 1.2/4.1 ± 1.6	0, 12 weeks	T1DM
Scalzo^29^	Sulphonylurea (Glimepiride)	DPP‐4i (Sitagliptin)	13/14	57 ± 3/59 ± 2	0.5	7.8 ± 0.2/7.6 ± 0.3	33.6 ± 1.4/32.3 ± 1.0	12	USA	10.1 ± 1/11.6 ± 1.1	−0.3 ± 0.9/0.2 ± 1.1	0, 12 weeks	T2DM
Martin^30^	DPP‐4i (Vildagliptin)	Sulphonylurea (Glibenclamide)	24/24	60.5 ± 7.0/59.4 ± 8.2	0.3	8.3 ± 1.0/7.9 ± 0.9	31.5 ± 3.3/30 ± 3.5	24	Brazil	8.6 ± 1.2/8.5 ± 1.3	−0.1 ± 1.2/−0.4 ± 1.3	0, 24 weeks	T2DM
Sofer^31^	Metformin	Placebo	32/31	51.9 ± 10.9/55.2 ± 14.0	0.5	NR	32.6 ± 5.8/31.5 ± 5.6	12	Israel	6.7 ± 1.1/6.3 ± 1.0	−1.0 ± 0.9/0.2 ± 1.0	0, 12 weeks	NAFLD
Watanabe^32^	TZD (Pioglitazone)	Sulphonylurea (Glibenclamide)	13/14	62.9 ± 10.3/65.1 ± 8.1	0.9	6.9 ± 0.2/7.2 ± 0.5	24.4 ± 4.4/24.7 ± 3.7	24	Japan	15.2 ± 2.5/15.2 ± 1.7	−1.0 ± 2.2/0.1 ± 1.8	0, 24 weeks	T2DM

*Note*: Data are expressed as the mean ± SD values.

Abbreviations: BMI, body mass index; CAD, coronary artery disease; CG, control group; CHF, chronic heart failure; DPP‐4 inhibitor, dipeptidyl peptidase‐4 inhibitor; EG, experience group; GLP‐1 RA, glucagon‐like peptide‐1 receptor agonist; NAFLD, non‐alcoholic fatty liver disease; NR, not reported; PWV, pulse wave velocity; SGLT‐2i, sodium‐glucose co‐transporter 2 inhibitor; T1DM, type 1 diabetes mellitus; T2DM, type 2 diabetes mellitus pre‐T2DM, type 2 diabetes mellitus; TZD, thiazolidinedione; ΔPWV, PWV change from baseline to post treatment.

### 3.2 Risk of bias quality assessment

**FIGURE 3:**
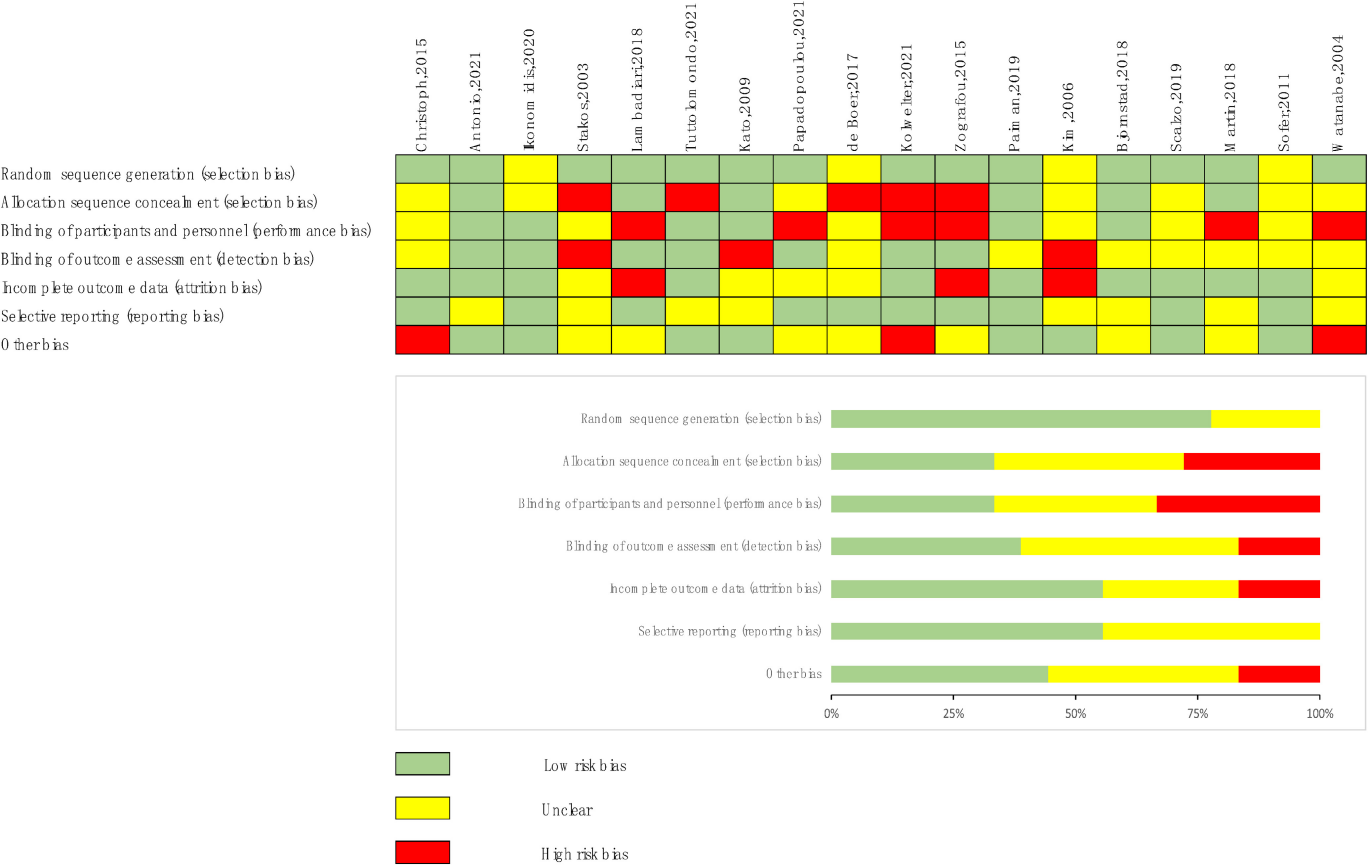
ROB assessment of all studies. ROB, risk of bias.

The qualities of the included studies were evaluated using the Cochrane Collaboration tool for risk of bias assessment. The method of participant and researcher blinding was not specified in six studies.^19^, ^22^, ^24^, ^25^, ^30^, ^32^ Three studies were regarded as “other bias” because the sex ratio was unbalanced (male:female ≥0.8).^15^, ^24^, ^32^ The details of the risk of bias quality assessment for each RCT are shown in Figure 3.

### 3.3 Primary outcome

**FIGURE 4:**
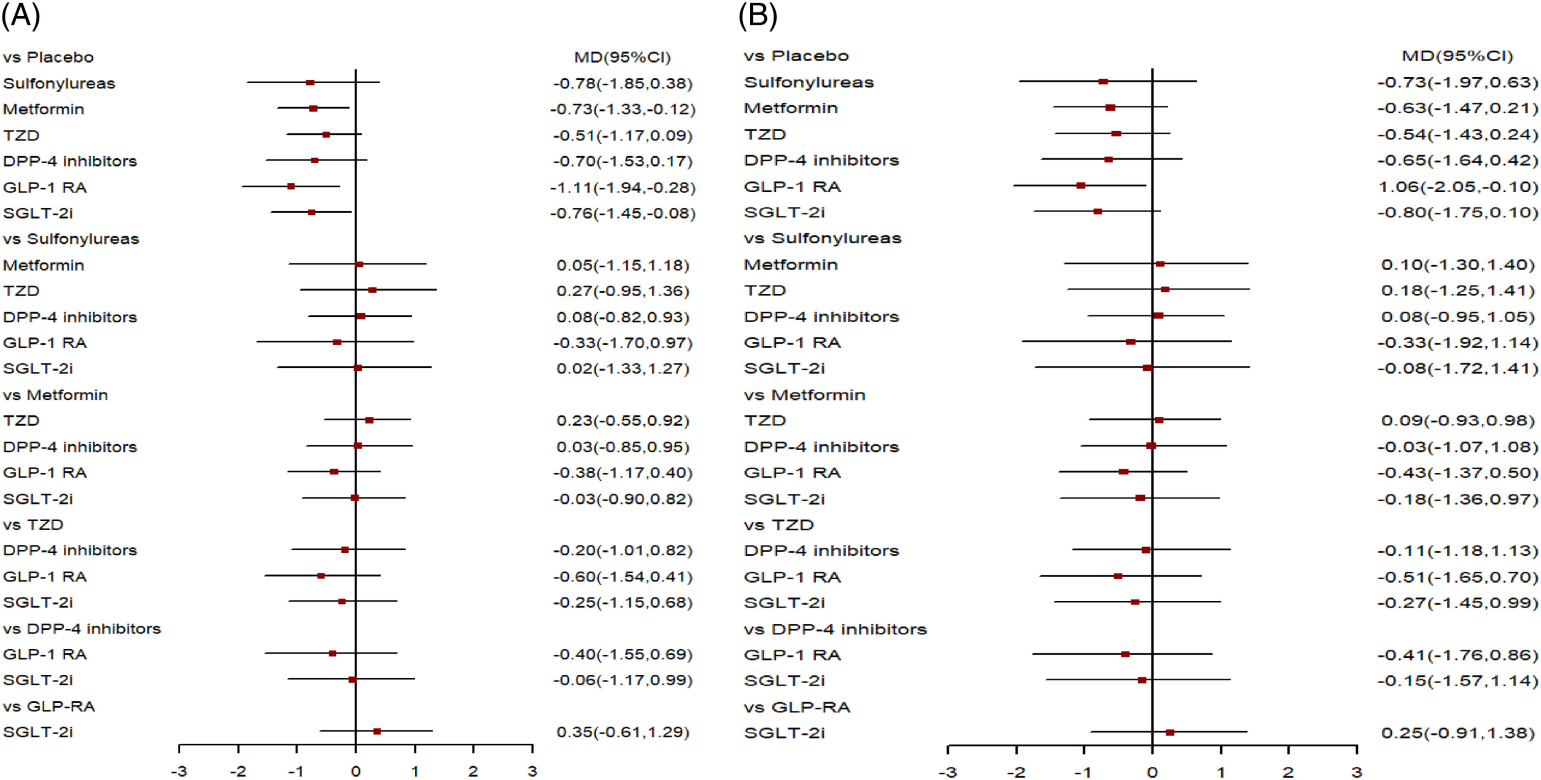
(A) Forest plot of comparison in all studies. (B) Forest plot of comparison in AGMD studies. AGMD, abnormal glucose metabolism diseases; CI, confidence interval; DPP‐4i, dipeptidyl peptidase‐4 inhibitor; GLP‐1 RA, glucagon‐like peptide‐1 receptor agonist; MD, mean difference; SGLT‐2i, sodium‐glucose co‐transporter 2 inhibitor; TZD, thiazolidinedione.

The primary outcome was ΔPWV in all 18 studies. Table 2 showed the network meta‐analysis results. Three classes of drugs showed significant benefits in terms of decreased PWV compared with placebo treatment: GLP‐1R agonists (MD = −1.11, 95% CI: −1.94 to −0.28), metformin (MD = −0.73, 95% CI: −1.33 to −0.12), and SGLT‐2 inhibitors (MD = −0.76, 95% CI: −1.45 to −0.08; Table 2). The forest plot of all comparison was showed in Figure 4A.

**TABLE 2:** Network meta‐analysis results for ΔPWV in all studies (18 trials, left lower half) and AGMD studies (15 trials, right upper half).

GLP‐1 RA	−0.34 (−1.92 to 1.14)	0.25 (−0.91 to 1.38)	−0.43 (−1.37 to 0.50)	−0.41 (−1.76 to 0.86)	−0.51 (−1.65 to 0.70)	**−1.06 (−2.05 to − 0.10)**
−0.33 (−1.70 to 0.97)	Sulfonylureas	−0.08 (−1.72 to 1.41)	0.10 (−1.30 to 1.40)	0.08 (−0.95 to 1.05)	0.18 (−1.25 to 1.41)	−0.73 (−1.97 to 0.63)
0.35 (−0.61 to 1.29)	0.02 (−1.33 to 1.27)	SGLT‐2i	−0.18 (−1.36 to 0.97)	−0.15 (−1.57 to 1.14)	−0.27 (−1.45 to 0.99)	−0.80 (−1.75 to 0.10)
−0.38 (−1.17 to 0.40)	0.05 (−1.15 to 1.18)	−0.03 (−0.90 to 0.82)	Metformin	−0.03 (−1.07 to 1.08)	0.09 (−0.93 to 0.98)	−0.63 (−1.47 to 0.21)
−0.40 (−1.55 to 0.69)	0.08 (−0.82 to 0.93)	−0.06 (−1.17 to 0.99)	0.03 (−0.85 to 0.95)	DPP‐4i	−0.11 (−1.18 to 1.13)	−0.65 (−1.64 to 0.42)
−0.60 (−1.54 to 0.41)	0.27 (−0.95 to 1.36)	−0.25 (−1.15 to 0.68)	0.23 (−0.55 to 0.92)	−0.20 (−1.01 to 0.82)	Thiazolidinedione	−0.54 (−1.43 to 0.24)
**−1.11 (−1.94 to −0.28)**	−0.78 (−1.85 to 0.38)	**−0.76 (−1.45 to −0.08)**	**−0.73 (−1.33 to −0.12)**	−0.70 (−1.53 to 0.17)	−0.51 (−1.17 to 0.09)	Placebo

*Note*: Treatments results are reported in league table. Significant pairwise comparisons of ΔPWV (PWV change from baseline to post treatment) are highlighted in dark gray boxes and underlined. Treatments estimates are MDs (mean, val2.5pc to val97.5pc) of the column‐defining treatment compared with the row‐defining treatment for ΔPWV. Mean differences (MDs) lower than 0 favor the column‐defining treatment, MDs higher than 0 favor the row‐defining treatment.

Abbreviations: AGMD, abnormal glucose metabolism disease; DPP‐4i, dipeptidyl peptidase‐4 inhibitor; GLP‐1 RA, glucagon‐like peptide‐1 receptor agonist; PWV, pulse wave velocity; SGLT‐2i, sodium‐glucose co‐transporter 2 inhibitor.

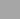
 AGM studies.

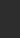
 Significant comparisons.

**FIGURE 5:**
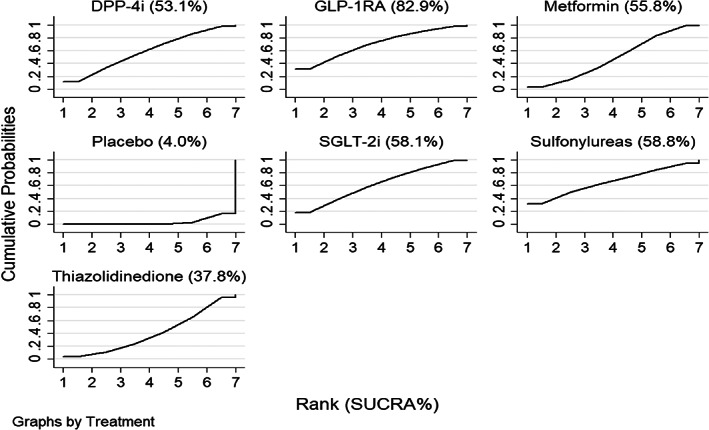
Rankings to improve PWV based on SUCRA curve. DPP‐4i, dipeptidyl peptidase‐4 inhibitor; GLP‐1 RA, glucagon‐like peptide‐1 receptor agonist; PWV, pulse wave velocity; SGLT‐2i, sodium‐glucose co‐transporter 2 inhibitor; SUCRA, the surface under the cumulative ranking curve.

The SUCRA curves show the detailed ranking of each treatment (Figure 5). Based on the SUCRA values, GLP‐1R agonists (SUCRA = 82.9%) showed the greatest ability to reduce PWV, followed by sulfonylureas (SUCRA = 58.8%), SGLT‐2 inhibitors (SUCRA = 58.1%), metformin (SUCRA = 55.8%), and DPP‐4 inhibitors (SUCRA = 53.1%). Although sulfonylureas demonstrated a high SUCRA among the six classes of antidiabetic drugs, this class of drugs did not demonstrate significant benefits in terms of reducing PWV compared with placebo treatment (MD = −0.78, 95% CI: −1.85 to 0.38; Table 2). The ability of thiazolidinediones (SUCRA = 22.7%) to reduce PWV was lower than the ability of any other antidiabetic drug, followed by the ability of placebo (SUCRA = 4.0%). The SUCRA values of all included studies were listed in Table 3.

**TABLE 3:** The SUCRA values of PWV change in drugs.

Treatment	SUCRA (95% CI)
All studies (*n* = 18)	
GLP‐1 RA	82.9% (0.17, 1.00)
Sulfonylureas	58.8% (0.00, 1.00)
SGLT‐2i	58.1% (0.17, 1.00)
Metformin	55.8% (0.17, 1.00)
DPP‐4i	53.1% (0.00, 1.00)
Thiazolidinediones	22.7% (0.00, 1.00)
Placebo	4.0% (0.00, 0.33)
Subgroup studies (*n* = 15)	
GLP‐1 RA	79.2% (0.17, 1.00)
Sulfonylureas	56.5% (0.00, 1.00)
SGLT‐2i	51.4% (0.00, 1.00)
Metformin	49.3% (0.00, 1.00)
DPP‐4i	56.3% (0.00, 1.00)
Thiazolidinediones	44.0% (0.00, 1.00)
Placebo	27.4% (0.00, 0.50)

*Note*: SUCRA, the surface under the cumulative ranking curve. Subgroup, abnormal glucose metabolism disease group.

Abbreviations: DPP‐4i, dipeptidyl peptidase‐4 inhibitor; GLP‐1 RA, glucagon‐like peptide‐1 receptor agonist; PWV, pulse wave velocity; SGLT‐2i, sodium‐glucose co‐transporter 2 inhibitor; SUCRA, surface under the cumulative ranking curve.

### 3.4 Subgroup outcomes

The AGMD group included 15 studies. In this subgroup, GLP‐1R agonists continued to exhibit an ability to reduce PWV compared with placebo (MD = −1.06, 95% CI: −2.05 to −0.10). However, SGLT‐2 inhibitors and metformin did not show a significant ability to reduce PWV compared with placebo (MD = 0.25, 95% CI: −0.91 to 1.38 and MD = −0.43, 95% CI: 1.37–0.50). The forest plot of comparison in AGMD group was shown in Figure 4B.

The SUCRA values in abnormal glucose metabolism studies also indicated that GLP‐1R agonists (SUCRA = 79.2%) had the greatest ability to reduce PWV compared with the other five classes of antidiabetic drugs. SGLT‐2 inhibitors (SUCRA = 62.6%) ranked second, followed by sulfonylureas, DPP‐4 inhibitors, and metformin with SUCRA values of 56.5%, 51.4%, and 49.3%, respectively. Thiazolidinediones (SUCRA = 44.0%) exhibit the lowest ability to reduce PWV among all included antidiabetic drugs, followed by placebo (SUCRA = 7.0%). The SUCRA values of subgroup studies were listed in Table 3.

### 3.5 Bias and assessment of inconsistency

**FIGURE 6:**
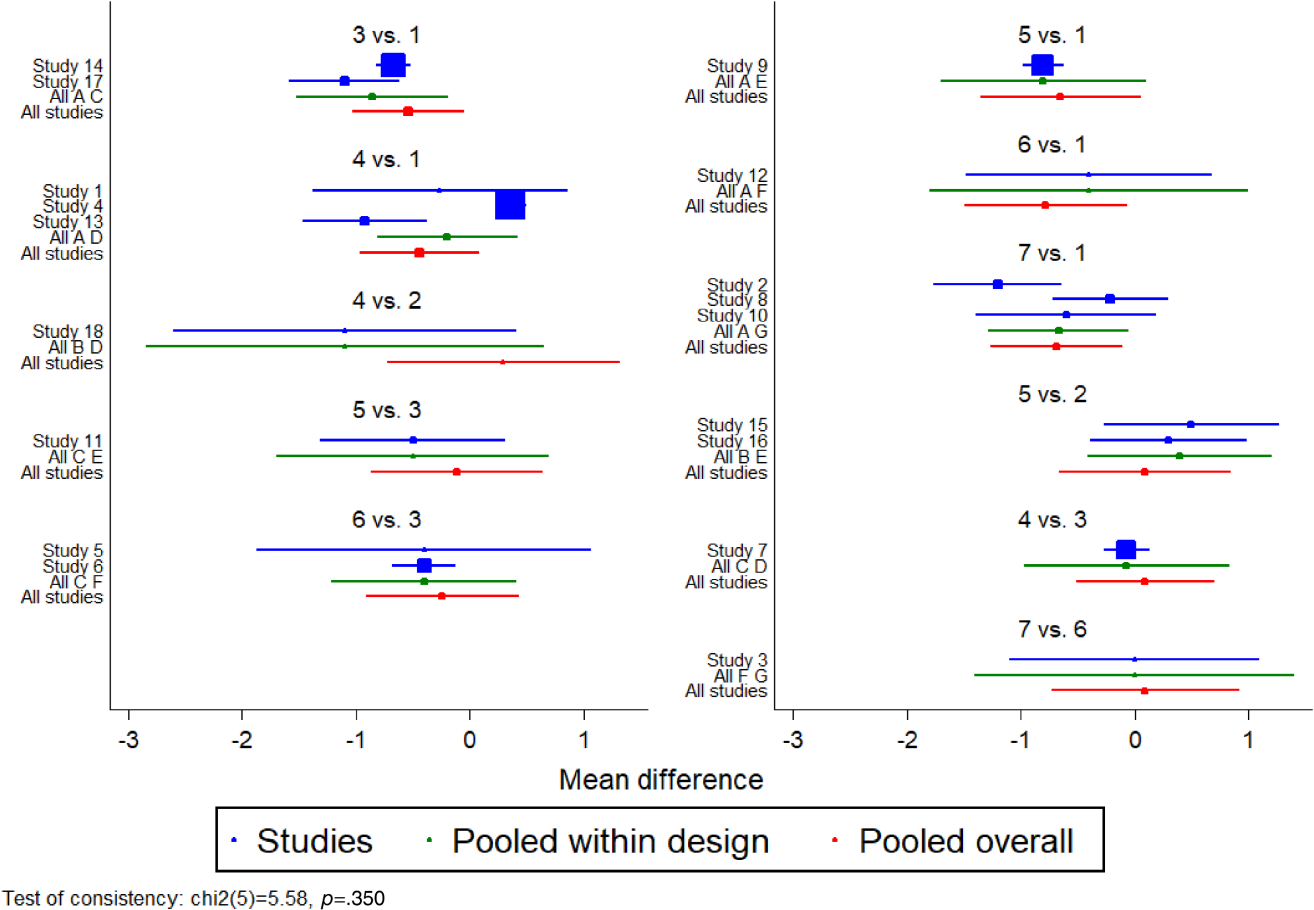
Global inconsistency of all studies. (A) Placebo (B) Sulfonylureas (C) Metformin (D) Thiazolidinedione (E) Dipeptidyl peptidase‐4 inhibitor (F) Glucagon‐like peptide‐1 receptor agonist (G) Sodium‐glucose co‐transporter 2 inhibitor.

**FIGURE 7:**
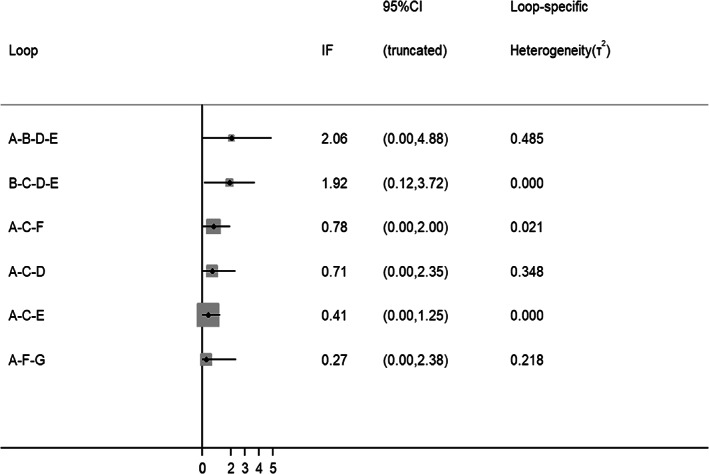
Loop consistency of all studies. (A) Placebo (B) Sulfonylureas (C) Metformin (D) Thiazolidinedione (E) Dipeptidyl peptidase‐4 inhibitor (F) Glucagon‐like peptide‐1 receptor agonist (G) Sodium‐glucose co‐transporter 2 inhibitor. IF, inconsistency factor.

**FIGURE 8:**
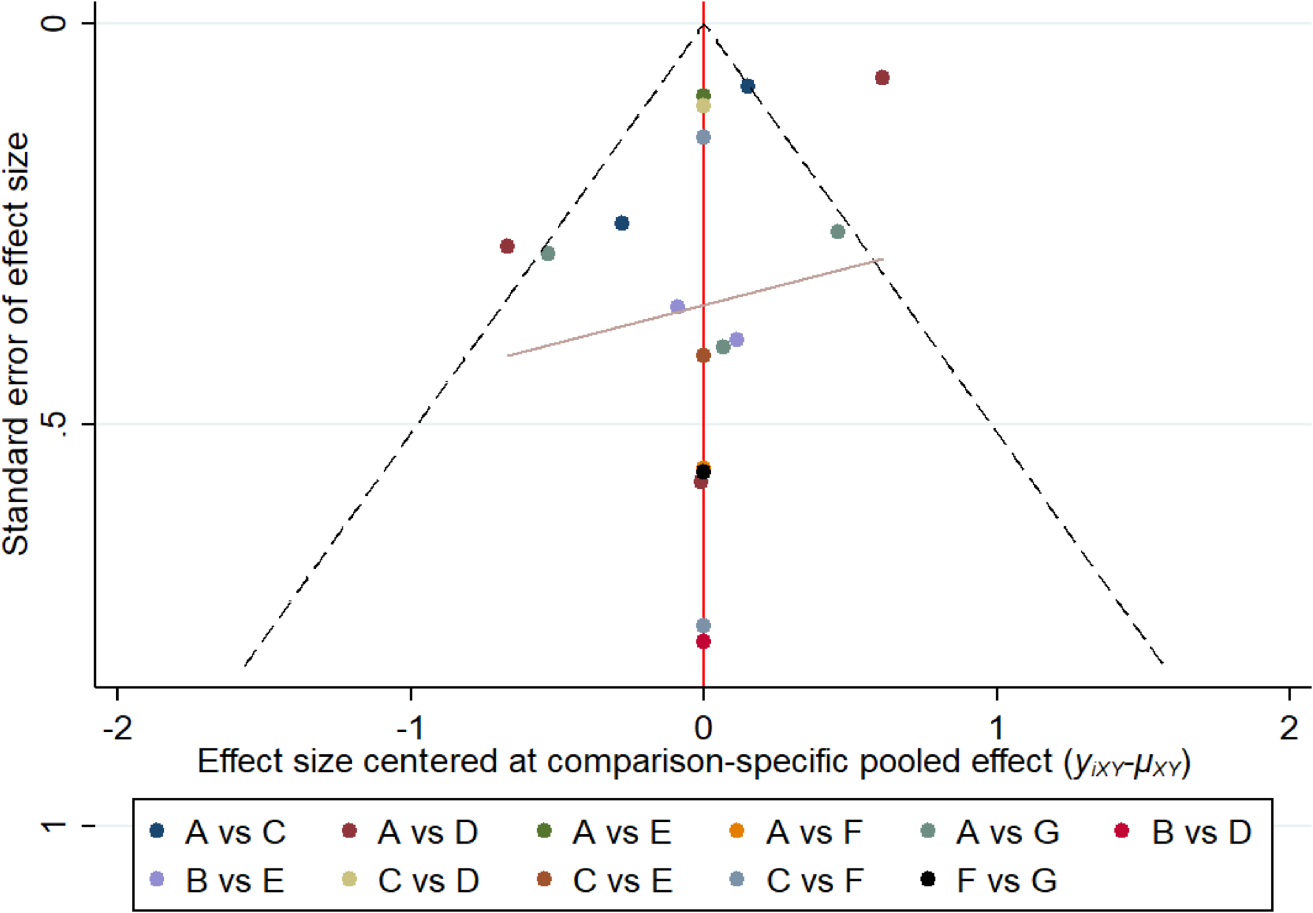
Funnel plot of all studies. (A) Placebo (B) Sulfonylureas (C) Metformin (D) Thiazolidinedione (E) Dipeptidyl peptidase‐4 inhibitor (F) Glucagon‐like peptide‐1 receptor agonist (G) Sodium‐glucose co‐transporter 2 inhibitor.

Significant inconsistencies were not identified in terms of the global inconsistency between direct and indirect estimates compared with all studies (*p* = .350, Figure 6). The loop consistency test showed the 95% CIs of six loops included 0 or nearly 0, indicating no significant inconsistency among any studies (Figure 7). Publication bias was investigated using visual examination of the funnel plot; several scatter plots showed nonsymmetrical inverted funnels (Figure 8).

## 4 DISCUSSION

In total, 18 RCTs were included in the NMA. GLP‐1R agonists, SGLT‐2 inhibitors, and metformin significantly decreased PWV compared with placebo, indicating these three drugs may have positive effects on arterial stiffness. In subgroup analysis, GLP‐1R agonists were the only class of drugs that demonstrated effects in patients with abnormal glucose metabolism, emphasizing the ability of GLP‐1R agonists to improve arterial stiffness in these specific populations.

The positive effects of GLP‐1R agonists on the vascular system have been confirmed in previous studies. For example, multiple RCTs have demonstrated that GLP‐1R agonists exhibit positive effects on endothelial function indicators, such as the reactive hyperemia index and flow‐mediated dilatation.^29^, ^33^ Similar to the potentially positive effect of endothelium protection, our NMA indicated that GLP‐1R agonists had the greatest positive effect on arterial stiffness among the six classes of antidiabetic drugs. Furthermore, GLP‐1R agonists were the only class of hypoglycemic drugs that significantly improved arterial stiffness in patients with abnormal glucose metabolism. The improvement of arterial stiffness with GLP‐1R agonists may be the result of comprehensive effects on the cardiovascular system. For example, GLP‐1R produced greater reduction of central systolic blood pressure and greater release of left ventricular myocardial strain.^30^, ^34^ Furthermore, GLP‐1R agonists inhibited high‐glucose‐induced inflammation and oxidative stress production in animal models; these positive vascular effects may be associated with improvements in lipid metabolism and weight, thereby reducing vascular adipose tissue‐derived inflammation.^35^, ^36^ These results may explain the positive effects of GLP‐1R agonists on vascular function under impaired glucose conditions.

Two large‐scale RCTs on newer classes of hypoglycemic agents, the Empagliflozin Outcome Trial in Patients with Chronic Heart Failure with Reduced Ejection Fraction (EMPEROR‐Reduced) and the Dapagliflozin and Prevention of Adverse‐outcomes in Heart Failure trial (DAPA‐HF), confirmed the protective effects of SGLT‐2 inhibitors on cardiovascular function and renal outcomes.^37^, ^38^ Our analysis showed that SGLT‐2 inhibitors significantly improved arterial stiffness compared with conventional antidiabetic agents, such as metformin and thiazolidinediones. The potential mechanisms by which SGLT‐2 inhibitors decrease PWV include reducing blood pressure, suppressing atherosclerosis, and promoting weight loss. Among the common hypoglycemic drugs, SGLT‐2 inhibitors are effective for weight loss,^39^ which is directly associated with reduced PWV and improved arterial compliance.^40^

Our NMA also showed that metformin decreased PWV. Metformin is a conventional insulin sensitizer that has been used for decades; it is a first‐line drug for endocrine metabolic abnormalities that has demonstrated multiple beneficial effects on CVD. The inhibition of adenosine monophosphate‐activated protein kinase (AMPK) activation may play a key role in the ability of metformin to alleviate endothelial impairment caused by abnormal glucose metabolism.^41^ Metformin also enhances vascular smooth muscle relaxation through AMPK activation; this process affects PWV.^42^ Additionally, metformin was the only treatment in our study with an ability to reduce PWV in NAFLD patients compared with placebo, suggesting that it may improve arterial stiffness in other metabolic disorders.

DPP‐4 inhibitors constitute another new class of antidiabetic drugs widely used in clinical practice; contrary to expectations, these drugs did not show a significant ability to reduce PWV. In a short‐term study, researchers observed a rapid improvement of arterial wall elasticity during treatment with the DPP‐4 inhibitor linagliptin. However, after 4 weeks of treatment, the PWV nearly returned to baseline, indicating that the effect of linagliptin on arterial stiffness is unstable after prolonged administration. Because arterial repair may require prolonged treatment, only long‐term trials were included in the present study to complement this analysis; the long‐term effect of DPP‐4 inhibitors on arterial stiffness was indeed limited. However, the protective effects of DPP‐4 inhibitors on vascular function, such as endothelial function, were confirmed in previous studies.^43^, ^44^

The other two classes of conventional antidiabetic drugs, sulfonylureas and thiazolidinediones, had limited effects on arterial stiffness. These two classes of drugs were used as second‐line antidiabetic drugs in clinical practice. There have been conflicting reports concerning the abilities of these two classes of drugs to protect against CVD. Because of their low SUCRA rank among the six classes of antidiabetic drugs in the present study, the effects of thiazolidinediones on PWV may remain negative. Christoph et al and Stakos et al found that the PWV values in thiazolidinedione groups were increased compared with baseline after 36 and 48 weeks of follow‐up, respectively.^29^, ^30^ Furthermore, thiazolidinediones did not reduce PWV in T2DM patients with metabolic syndrome during 12 weeks of observation, indicating that arterial stiffness was not improved in this metabolism‐related disease. Similarly, despite the higher SUCRA rank of sulfonylureas compared with SGLT‐2 inhibitors and metformin, sulfonylureas did not exhibit a significant ability to improve arterial stiffness. The potential side effects of these two classes of drugs include weight gain and fluid retention, which may burden the circulatory system; such effects may explain the limited abilities of these two drugs to improve arterial stiffness.

In this NMA, the effects of six classes of antidiabetic drugs on arterial stiffness were summarized through assessment of PWV. Three classes of antidiabetic drugs had significant positive effects on arterial stiffness in all participants. GLP‐1 receptor agonists were the only class of antidiabetic drugs that showed specific positive effects on arterial stiffness in patients with abnormal glucose metabolism, indicating these novel antidiabetic drugs may have the ability to improve vascular function in patients with abnormal glucose metabolism.

## 5 LIMITATIONS

Several limitations should be considered when interpreting the results of this study. First, to ensure comparability in this study, a long‐term observation period (≥12 weeks) was used. Although evidence from short‐term studies was limited, the time frame for PWV measurement may have contributed to heterogeneity. Second, six classes of antidiabetic drugs were included in this study; however, treatments such as α‐glucosidase inhibitors or glinides were not included because they did not meet the inclusion criteria. Third, multiple factors may have contributed to the inconsistencies observed in this study. The PWV observation period in each study ranged from 12 to 48 weeks. Regional differences existed in 18 studies: 3 study populations were from Asia, 9 were from Europe, 4 were from North America, 1 was from South America, and 1 was from the Middle East. Finally, only published studies were included; thus, the possibility of publication bias could not be ruled out.

## 6 CONCLUSIONS

Three classes of antidiabetic drugs—GLP‐1R agonists, SGLT‐2 inhibitors, and metformin—have the potential to improve arterial stiffness. Among the six classes of antidiabetic drugs analyzed, GLP‐1R agonists were the only class of drugs to improve arterial stiffness in patients with abnormal glucose metabolism diseases.

## AUTHOR CONTRIBUTIONS

Jincheng Wang performed statistical analysis. Yuhan Wang wrote the manuscript. Yu Li and Xiaomin Fu conducted the database search, screened, and extracted data. Jiamei Zhang and Han Zhang interpreted the data for analysis. Zhiqin Guo, Ying Yang, and Kaining Kang contributed to the discussion and editing. Yueheng Wang designed the study. Wei Zhang, Li Tian, Yanqiang Wu revised the draft manuscript. The corresponding author Shuanli Xin and Hongzhou Liu had full access to the data and had final responsibility for the decision to submit for publication. All authors read and approved the final manuscript.

## FUNDING INFORMATION

There were no sources of funding for this research.

## CONFLICT OF INTEREST STATEMENT

The authors declare they have no conflicts of interests.

## Supporting information


**Appendix S1.** Research strategies.

## Data Availability

All data collected in this study are included in the published article.
