# Supplementary material for: Effects of first‐line antidiabetic drugs on the improvement of arterial stiffness: A Bayesian network meta‐analysis
Source: J Diabetes. 2023 May 10;15(8):685–98. doi: 10.1111/1753-0407.13405 (PMC10415870; doi:10.1111/1753-0407.13405)
Supplement: Supplementary file 1 — Appendix S1. Research strategies. [file JDB-15-685-s001.pdf]

# Research strategies

**Database: Searching PubMed, Embase, Cochrane.**

**PubMed search strategy**

#1 (((((Hypoglycemic Agents [Mesh]) OR (Hypoglycemic Agents)) OR (antidiabetic*)) OR (hypoglycemic*)) OR (hypoglycaemic*)) OR (antihyperglycemic*)

304,135

#2 ((((((((((((((Sodium-Glucose Transporter 2 Inhibitors[mesh]) OR (Sodium-Glucose Transporter 2 Inhibitor)) OR (Sodium-Glucose Transporter 2 Inhibitors)) OR (Sodium Glucose Transporter 2 Inhibitor)) OR (Sodium Glucose Transporter 2 Inhibitors)) OR (SGLT-2 Inhibitors)) OR (SGLT-2 Inhibitor)) OR (SGLT-2I)) OR (Gliflozins [Title/Abstract])) OR (Canaglifozin[Title/Abstract])) OR (Dapagliflozin[Title/Abstract])) OR (Sotagliflozin[Title/Abstract])) OR (Empagliflozin[Title/Abstract])) OR (Ertugliflozin[Title/Abstract])) OR (Tofogliflozin[Title/Abstract])) OR (Ipragliflozin[Title/Abstract])) OR (Remogliflozin[Title/Abstract])

7,347

#3 ((((((((((((((Dipeptidyl-Peptidase IV Inhibitors [Mesh]) OR (Dipeptidyl Peptidase IV Inhibitors)) OR (Dipeptidyl Peptidase IV Inhibitor)) OR (Dipeptidyl Peptidase 4 Inhibitors)) OR (Dipeptidyl Peptidase 4 Inhibitor)) OR (DPP-IV Inhibitors)) OR (DPP-IV Inhibitor)) OR (Gliptin[Title/Abstract])) OR (sitagliptin[Title/Abstract])) OR (saxagliptin[Title/Abstract])) OR (vildagliptin[Title/Abstract])) OR (linagliptin[Title/Abstract])) OR (gemigliptin[Title/Abstract]))) OR (canagliptin[Title/Abstract])) OR (teneligliptin[Title/Abstract]) OR (alogliptin[Title/Abstract])

9,567

#4 ((((((((((((Glucagon-Like Peptide 1 [MeSH]) OR (Glucagon Like Peptide 1)) OR (Glucagon-Like Peptide-1)) OR (Glucagon-like peptide-1 agonists)) OR ( (Glucagon-like peptide-1 agonist)) OR (GLP-1 agonists)) OR (GLP-1 agonist)) OR (GLP-1 RAs)) OR (GLP-1 RA)) OR (Exenatide [Title/Abstract])) OR (Liraglutide [Title/Abstract])) OR (Semaglutide [Title/Abstract])) OR (Dulaglutide [Title/Abstract])) OR (Lixisenatide

[Title/Abstract])

19,797

#5 ((((Metformin [mesh])) OR (Metformin [Title/Abstract])) OR (Metformin HCl [Title/Abstract])) OR (Metformin Hydrochloride [Title/Abstract])

26,834

#6 (((((Thiazolidinediones[mesh]) OR (Thiazolidinediones)) OR (Thiazolidinedione)) OR (TZD)) OR (Pioglitazone [Title/Abstract])) OR (Rosiglitazone [Title/Abstract])) OR (Troglitazone [Title/Abstract])

18,546

#7 (((((((((Sulfonylurea Compounds [mesh]) OR (Sulfonylureas)) OR (Sulfonylurea)) OR (Glibenclamide[Title/Abstract])) OR (Glyburide[Title/Abstract])) OR (Glibornuride[Title/Abstract])) OR (Gliclazide[Title/Abstract])) OR (Glimepiride[Title/Abstract])) OR (Glipizide[Title/Abstract])) OR (Gliquidone[Title/Abstract])) OR (Glycopyramide[Title/Abstract])

30,437

#8 (((((((Nateglinide [mesh]) OR (glinide)) OR (Repaglinide)) OR (Mitiglinide)) OR (Nateglinide)) OR (Mitiglinide))

1,669

#9 (((((((Glycoside Hydrolase Inhibitors [mesh]) OR (Acarbose [mesh])) OR (α-glucosidase inhibitors)) OR (α-glucosidase inhibitor)) OR (alpha glucosidase inhibitors)) OR (alpha glucosidase inhibitor) OR (Acarbose [Title/Abstract])) OR (Voglibose[Title/Abstract])) OR (Miglitol[Title/Abstract])) OR (AGI)

13,889

#10 (Vascular Stiffness[Mesh]) OR ( vascular Stiffness) OR (pulse wave analysis[mesh]) OR (arterial stiffness) OR (aortic stiffness) OR (pulse wave velocity) OR (blood pulse wave velocity) OR (PWV) OR cfPWV OR baPWV

364,727

#11 ((((((((Randomized Controlled Trial [Publication Type]) OR (Randomized)) OR (Randomised)) OR (Randomization))) OR (randomly))) OR

(placebo [Title/Abstract]))

1,616,351

#12 (#2 OR #3 OR #4 OR #5 OR #6 OR #7 OR #8 OR #9) AND #10 AND #11

214

**Embase search strategy**

#1 'crossover procedure':de OR 'double-blind procedure':de OR 'randomized controlled trial':de OR 'single-blind procedure':de OR random*:de,ab,ti OR factorial*:de,ab,ti OR crossover*:de,ab,ti OR ((cross NEXT/1 over*):de,ab,ti) OR placebo*:de,ab,ti OR ((doubl* NEAR/1 blind*):de,ab,ti) OR ((singl* NEAR/1 blind*):de,ab,ti) OR assign*:de,ab,ti OR allocat*:de,ab,ti OR volunteer*:de,ab,ti

2,931,605

#2 'sodium glucose cotransporter 2 inhibitor'/exp

#3 (‘sodium glucose cotransporter 2 inhibitors' OR ‘sodium glucose cotransporter 2 inhibitor' OR 'sglt-2 inhibitors' OR 'sglt-2 inhibitor' OR 'sodium-glucose transporter 2 inhibitors' OR 'sodium-glucose transporter 2 inhibitor' OR 'gliflozin' OR 'canaglifozin' OR 'dapagliflozin' OR 'sotagliflozin' OR 'empagliflozin' OR 'ertugliflozin' OR 'tofogliflozin' OR 'ipragliflozin' OR 'remogliflozin') ti,ab,kw

#2 OR #3

18,457

#4 'dipeptidyl peptidase iv inhibitor'/exp

#5 ('dipeptidyl peptidase iv inhibitor' OR 'dipeptidyl peptidase iv inhibitors' OR 'dipeptidyl-peptidase 4 inhibitors' OR 'dipeptidyl peptidase 4 inhibitors' OR 'inhibitors, dipeptidyl-peptidase iv' OR 'dpp-iv inhibitor' OR 'gliptin' OR 'sitagliptin' OR 'saxagliptin' OR 'vildagliptin' OR 'linagliptin' OR 'gemigliptin' OR 'canagliptin' OR 'teneligliptin' OR 'alogliptin') ti,ab,kw

#4 OR #5

24,276

#6 'glucagon like peptide 1 receptor agonist'/exp

#7 ('glucagon like peptide 1' OR 'glucagon-like peptide-1' OR 'glucagon-like peptide-1 agonists' OR 'glucagon like peptide 1 receptor agonist' OR 'glp-1 agonists' OR 'glp-1 agonist' OR 'glp-1 ras' OR 'glp-1 ra' OR 'exenatide' OR 'liraglutide' OR 'semaglutide' OR 'dulaglutide' OR 'lixisenatide') ti,ab,kw

#6 OR #7

43,397

#8 '2,4 thiazolidinedione derivative'/exp

#9 ('2,4 thiazolidinedione derivative' OR 'thiazolidinediones' OR 'thiazolidinedione ' OR 'tzd' OR 'pioglitazone' OR 'rosiglitazone' OR 'troglitazone') ti,ab,kw

#8 OR #9

31,564

#10 'metformin'/exp

#11 ('metformin' OR 'metformin hcl OR 'metformin hydrochloride') ti,ab,kw

#10 OR #11

53,883

#12 'glycosidase inhibitor'/exp

#13 ('glycosidase inhibitor' OR 'ɑ-glucosidase inhibitors' OR 'ɑ-glucosidase inhibitor' OR 'alpha glucosidase inhibitor' OR 'acarbose' OR 'miglitol' OR 'voglibose' OR 'alogiptin') ab,ti,kw

#12 OR #13

37,889

#14 'repaglinide' OR 'nateglinide' OR 'mitiglinide'/exp

#15 ('repaglinide' OR 'nateglinide' OR 'mitiglinide') ab,ti,kw

#14 OR #15

5,771

#16 'sulfonylurea'/exp

#17 ( 'sulfonylureas' OR 'sulfonylurea' OR 'glibenclamide' OR 'glyburide' OR 'glibornuride' OR 'gliclazide' OR 'glimepiride' OR 'glipizide' OR 'gliquidone' OR 'glycopyramide' ) ab,ti,kw

#16 OR #17

40,095

#18 'antidiabetic agent'/exp

#19 ('hypoglycemic agents' OR antidiabetic* OR hypoglycemic* OR antihyperglycemic*) ti,ab,kw

#18 OR #19

582,190

#20 ‘arterial stiffness' OR 'pulse wave'/exp

#21 ('vascular Stiffness':ab,ti,kw OR 'arterial stiffness':ab,ti,kw OR 'aortic stiffness':ab,ti,kw OR 'pulse wave velocity':ab,ti,kw OR 'blood pulse wave velocity':ab,ti,kw

#20 OR #21

44,940

#1 AND (#2 OR #3 OR #4 OR #5 OR #6 OR #7 OR #8 OR #9 OR #10 OR #11 OR #12 OR #13 OR #14 OR #15 OR #16 OR #17 OR #16 OR #17 OR#18 OR #19) AND (#20 OR #21)

560

**Cochrane search strategy**

#1 MeSH descriptor: [Sodium-Glucose Transporter 2 Inhibitors] explode all trees

#2 (Sodium-Glucose Transporter 2 Inhibitor OR Sodium Glucose Transporter 2 Inhibitor OR SGLT-2 Inhibitor OR Canaglifozin OR Dapagliflozin OR Sotagliflozin OR Empagliflozin OR Ertugliflozin OR Tofogliflozin OR Ipragliflozin OR Remogliflozin):ti,ab,kw

#1 OR #2

3,479

#3 MeSH descriptor: [Dipeptidyl-Peptidase IV Inhibitors] explode all trees

#4 (Dipeptidyl-Peptidase 4 Inhibitors OR Dipeptidyl Peptidase 4 Inhibitors OR Inhibitors, Dipeptidyl-Peptidase IV OR DPP-IV Inhibitor OR sitagliptin OR saxagliptin OR vildagliptin OR linagliptin OR gemigliptin OR canagliptin OR alogliptin):ti,ab,kw

#3 OR #4

4,312

#5 MeSH descriptor: [Glucagon-Like Peptide 1] explode all trees

#6 (Glucagon-Like Peptide 1 OR Glucagon Like Peptide 1 OR Glucagon-Like Peptide-1 OR Glucagon-like peptide-1 agonists OR GLP-1 agonist OR GLP-1 agonists OR GLP-1 RAs OR Exenatide OR Liraglutide OR Semaglutide OR Dulaglutide OR Lixisenatide):ti,ab,kw

#5 OR #6

6,886

#7 MeSH descriptor: [Thiazolidinediones] explode all trees

#8 (Thiazolidinediones OR Thiazolidinedione OR TZD OR Pioglitazone OR Rosiglitazone OR Troglitazone):ti,ab,kw

#7 OR #8

4,134

#9 MeSH descriptor: [Metformin] explode all trees

#10 (Metformin OR Metformin HCl OR Metformin Hydrochloride):ti,ab,kw

#9 OR #10

11,712

#11 MeSH descriptor: [Sulfonylurea Compounds] explode all trees

#12 (Sulfonylurea OR Sulfonylureas OR Glibenclamide OR Glyburide OR Glibornuride OR Gliclazide OR Glimepiride OR Glipizide OR Gliquidone OR Glycopyramide):ti,ab,kw

#11 OR #12

4,885

#13 MeSH descriptor: [Glycoside Hydrolase Inhibitors] explode all trees

#14 MeSH descriptor: [Acarbose] explode all trees

#15 (α-glucosidase inhibitors OR α-glucosidase inhibitor OR alpha glucosidase inhibitor OR Acarbose OR Miglitol OR Voglibose OR AGI):ti,ab,kw

#13 OR #14 OR #15

3061

#16 MeSH descriptor: [Nateglinide] explode all trees

#17 (Repaglinide OR Nateglinide OR Mitiglinide OR Glinides OR Glinide):ti,ab,kw

#16 OR #17

745

#18 MeSH descriptor: [vascular Stiffness] explode all trees

#19 MeSH descriptor: [pulse wave analysis] explode all trees

#20 (vascular Stiffness) OR (arterial stiffness) OR (aortic stiffness) OR (pulse wave velocity) OR (blood pulse wave velocity) OR (PWV):ti,ab,kw

#18 OR #19 OR #20

4834

(#1 OR #2 OR #3 OR #4 OR #5 OR #6 OR #7 OR #8 OR #9 OR #10 OR #11 OR #12 OR #13 OR #14 OR #15 OR #16 OR #17) AND (#18 OR #19 OR #20)

345
